# Highlighting “Risky Remands” Through Prisoner Death Investigations: People With Very Severe Mental Illness Transitioning From Police and Court Custody Into Prison on Remand

**DOI:** 10.3389/fpsyt.2022.862365

**Published:** 2022-04-01

**Authors:** Philippa Tomczak

**Affiliations:** prisonHEALTH Research Group, School of Sociology and Social Policy, University of Nottingham, Nottingham, England

**Keywords:** pre-trial detention, liaison and diversion, mental capacity, fitness to plead, prison oversight, death investigation, remand

## Abstract

Prison suicide/self-inflicted death is an international public health crisis, harming stakeholders including bereaved families, prisoners, prison staff and death investigators. England and Wales' record prison suicide numbers in 2016 cost at least £400 million. Death rates are an indicator of prison safety, and unsafe prisons mean unsafe societies. I present four case studies of people with very severe mental illness who were remanded to prison from police and/or court custody and went on to take their own lives in prison. I use publicly available data from Ombudsman and Coronial death investigations in England and Wales, highlighting that these accessible sources could be more widely mobilized to reduce the substantial harms and costs of prisoner deaths. Case studies include three men (Lewis Francis, Jason Basalat and Dean Saunders) and one woman (Sarah Reed) who took their own lives between January 2016 and April 2017. All four people were clearly very mentally unwell at the time of their alleged offense and remand to prison. I develop the concept of “risky remands” to highlight that people with very severe mental illness being remanded to prison is a particularly problematic practice. I highlight the implications of people with very severe mental illness transitioning into prison in the first place, arguing that being remanded to prison is not an acceptable or safe pathway into healthcare. I illustrate that police custody suites and courts may lack awareness of mechanisms and/ or the practical ability to transfer ill detainees charged with a serious crime to mental health facilities for assessment and/ or treatment. My analysis amplifies and extends recent Criminal Justice Joint Inspection findings that it is unacceptable to use prisons as a “place of safety,” and that the Department of Health and Social Care, NHS England and the Welsh Government must increase the supply of medium and high secure beds. Moreover, Ombudsman investigations did not engage with the remand transition, effectively legitimizing this risky practice for very ill people. As such, my analysis also counters the apparent “problem of implementation” in prison oversight, instead questioning what reviewers recommend, based on which evidence.

## Introduction

Prison suicide/ self-inflicted death^1^ is a public health and ethical concern reaching crisis proportions around the world (2, 3). Prisoner mortality rates are up to 50% higher than rates in comparable communities outside [(4): p. 9]. Rates of suicide amongst prisoners are consistently elevated relative to people of similar age and sex in the community (5). Prison suicides cause (enduring) harm across stakeholders including bereaved families, prisoners, prison staff and death investigators, and negatively affect staff wellbeing, absence and prison regimes (6–9). Suicides in locales can also directly and indirectly lead to further deaths through “clustering” (10), potentially compounding the risks, harms and costs of each death. “Clustering” can result from changes to prisoner behavior, regime disruptions and changes in staff practice such as increased fear and risk aversion after each death (9, 11).

England and Wales saw record prison suicides in 2016, which consumed around £400 million of public funds (6). Ministry of Justice Safety in Custody statistics^2^ demonstrate that deaths, suicides, self-harm and assaults all increased after the 2012 introduction of benchmarking policies, which reduced staffing levels in public prisons by ~25% (12). Death rates are an indicator of prison safety, and unsafe prisons mean unsafe societies (13). Poorer prisoner health (14) and poorer prison social climates (15) correlate with higher reoffending rates. Reoffending in England and Wales costs £18.1 billion annually, whilst creating new harms (e.g., trauma) daily (16). In this article, I present four case studies of people with very severe mental illness who transitioned from police and/or court custody into prison on remand (before trial) and went on to take their own lives in prison. Lewis Francis, Jason Basalat, Sarah Reed and Dean Saunders died by their own hand between January 2016 and April 2017. Remand prisoners are generally at an elevated risk of suicide amongst prison populations (17) and comprise a substantive 30% of the 11 million people imprisoned globally (18), whose overall mortality rates are significantly above those amongst comparators in the community (4). However, the four people in these case studies were clearly very mentally unwell at the time of their alleged offense and remand, making their remand to prison particularly problematic and risky. I view people with very severe mental illness at the time of their alleged offense and remand to prison as qualitatively different from prisoners who develop severe mental illness whilst in prison.

Legal theorists have repeatedly argued that criminal responsibility should only be imposed on individuals who have the capacity and freedom to choose how they behave (*doli (in)capax*) (19). Human capacity for rationality (*mens rea*) is key to forming intent and underpins criminal responsibility in theory (20). Despite the fundamental importance of the doctrine of competence to stand trial, which is known as “fitness to plead” in English law, this principle is more challenging to uphold in practice. A startlingly low number of defendants are adjudicated unfit to plead in England and Wales annually (21). According to (22) “findings of ‘unfitness' are so rare that there is considerable professional unease” with the current assessment process, and as such “mentally disordered defendants may be subjected unfairly to criminal trials” and indeed punishment. In English law, the test for fitness arises from case law in *R v Pritchard* (1836) 7 C & P 303, 173 ER 135. This narrow, archaic law is widely regarded as in need of reform to be fit for purpose and protect vulnerable defendants in the twenty first century (21, 23). Yet, people with (serious) mental illness and disabilities are overrepresented in the prison population, and too frequently convicted “in circumstances where their real responsibility for the crime at issue is at best questionable” [(24): p. 776]. In practical terms, the defense may not raise the issue of fitness to plead until the Plea and Trial Preparation Hearing, which takes 4 to 6 weeks, during which the unwell defendant may be remanded in custody [(25): p. 81]. This is problematic as, although imprisonment is too frequently “tacitly accepted” as a substitute for treatment in hospital, “the prison is not a safe place to wait for a hospital bed” [(26): p. 5-6].

Scholars across disciplines recognize that deinstitutionalisation of mental health treatment has led to its substitution with imprisonment [e.g., (27)]. The costs of “misplaced” patients in the criminal justice system have been recognized in various reports e.g., the 1992 *Reed Report* and the 2009 *Bradley Report:* which designated failures to address mental health needs as a fundamental cause of chronic dysfunction in the criminal justice system and stimulated various liaison and diversion initiatives nationally (28). In England and Wales, the National Health Service and criminal justice system now have joint responsibility for diverting people from prison if themselves and wider society are better served by addressing underlying health needs, including mental health problems [(29): p. 1005]. However, the recent Criminal Justice Joint Inspection “highlights some disappointing findings and makes clear that not enough progress has been made in the 12 years since the Bradley Review” [CJJI (25): p. 4]. Whilst the national development of liaison and diversion services has brought some improvements, their success is dependent on the varying local availability of mental health services and beds that people can be diverted to (30, 31). Liaison and diversion outcomes also vary with perpetrator characteristics, for example males and those with a longer history of criminalization were more likely to receive custodial sentences than be diverted to healthcare (31).

Detention settings such as police cells and prisons are “not a particularly humane place to detain someone with a mental disability” [(24): p. 774]. In England and Wales, the recent Criminal Justice Joint Inspection (CJJI) [(25): p. 3] highlighted that “the criminal justice process itself, for example the experience of custody, can have a severe and negative impact on someone's mental health, particularly if they are already suffering a mental illness.” Indeed, in the case of *MS v. UK (2012)* (application 24527/ 08), the European Court of Human Rights ruled that holding MS in police detention without adequate medical care for over 72 h under section 136 of the *Mental Health Act 1983*, whilst the police and mental health services were unable to agree on alternative accommodation, constituted a breach of Article 3 (freedom from torture and inhuman or degrading treatment) of the European Convention on Human Rights/ *Human Rights Act 1998* in the UK. Degrading treatment is particularly pertinent in cases where people are vulnerable or suffering from ill-health and refers to humiliating and undignified actions, such as prolonged interrogation and detention (32). The Irish Office of the Inspector of Prisons recently noted [(33): p. 4], prisons “are not, and cannot be considered therapeutic environments for the provision of mental health care and treatment.” Moreover, there are high levels of psychiatric morbidity amongst prisoners (34) and many prison staff are not trained in mental health work (35, 36). Despite developments over the last two decades, prison mental health in-reach services fall short of community equivalence and have high caseloads, with each nurse covering around 500 prisoners and each doctor over 3,700 prisoners, whilst 24 h psychiatric cover is uncommon (34). Even at its best, the containment-oriented criminal justice system can be considered anti-therapeutic for prisoners and is not a substitute for the care-oriented National Health Service (37). Substituting imprisonment for mental health care also has organizational implications across prisons, as managing people with severe mental illness has negative consequences for service delivery and staff stress, which have implications for all prisoners and staff (9, 38). Yet, unlike mental health facilities, prisons cannot refuse anyone sent to them, no matter how unsuitable the facilities (39).

Between cases of people committing minor offenses who can be diverted into community mental health treatment and people who are eventually found, or should be found, unfit to plead there is a gray area of people for whom release into the community is not appropriate for themselves and/or others, yet imprisonment for any period of time brings significant risks. From police custody, detainees can be transferred to hospital for treatment and or assessment under section 2 and / or 3 of the *Mental Health Act 1983*. Detainees can also be processed through criminal justice pathways and transition to court and prison. Compounding this gray area, definitional issues are problematic across the different criminal justice agencies. There is “no common definition of mental health used” across the different institutions of the criminal justice system, which “leads to individual's needs being missed as they progress through the system” [(25): p. 4]. The Crown Prosecution Service problematically state that some offenses are “too serious” for diversion from prison (40), indicating an urgent need to clarify the CPS's definition of diversion. These definitional issues across institutions are extended in the analysis section.

Amplifying and problematizing recent critique of the “totally unacceptable” practice that “prisons continue to be used as a place of safety” [(25): p. 10] and the recommendation that “the Department of Health and Social Care, NHS England and Improvement and Welsh Government should: ensure an adequate supply of medium and high secure beds” [(25): p. 12], I develop the concept of “risky remands,” highlighting that people with very severe mental illness being remanded to prison is a particularly risky practice requiring urgent reconsideration and multiagency action. I highlight the implications of people with very severe mental illness at the time of their offense and remand transitioning into prison *in the first place*, arguing that being remanded to prison is not an acceptable pathway into healthcare, and that discussions of hospital transfer times must not assume that prisoners will survive the series of criminal justice transitions that too frequently precede healthcare treatment.

Deaths also occur in hospitals, and we cannot know whether these four individuals would have survived if they had been detained in hospital rather than prison. However, the imprisonment of these very ill people is hard to defend, as psychiatric staffing and treatment in prison are in general not equivalent to that in the community (34–37). My focus on deaths of remand prisoners is important, as both deaths and remand prisoners risk being overlooked in evaluations and control measures. For example, Disley et al. [(31): p. vi] recent evaluation of the impact of the National Model for Liaison and Diversion on healthcare and criminal justice outcomes used reconviction measures, but those who die will not be reconvicted, and measured the likelihood of receiving a custodial sentence [(31): p. 78], which overlooks the remand transition pre-sentence. I also highlight Coroner's findings that police custody suites and courts may lack mechanisms to transfer detainees charged with a serious crime to mental health facilities and that Prisons and Probation Ombudsman (PPO) investigations did not engage with the remand transition, effectively legitimizing the risky practice of using prison as a “place of safety” and/ or acceptable pathway into secure hospital. As such, my analysis also counters the apparent “problem of implementation” in prison oversight, instead questioning what reviewers recommend, based on which evidence.

All deaths in state detention threaten the fundamental human right to life (41). (Inter)national human rights and humanitarian law impose obligations to protect life and “effectively” investigate suspected violations of the right to life (42, 43). In the 47 Council of Europe member states, all unexplained deaths in state detention automatically engage Article 2 of the European Convention on Human Rights, which includes a duty to “effectively” investigate potential violations of the right to life. Every prisoner death investigation provides a window to identify, organize and apply learning and resource gaps, that could enhance safety in prisons and societies. Death investigations are potentially significant triggers for harm reduction, although this potential is yet unrealized and has attracted little scholarship (44). Prisoner death investigation methodologies, evidence bases, findings and implications require development across legal frameworks, around the world (44, 45). My case study jurisdiction of England and Wales is particularly informative for analyzing prisoner death investigations because it has anomalously high imprisonment rates in Western Europe and has experienced recent dramatic declines in prison safety and record prison suicide numbers, whilst simultaneously claiming world-leading prison oversight expertise (13). In England and Wales, the PPO has, since 2004, assisted the Coroner's inquest to fulfill the investigative obligation arising under Article 2 [(46): p. 9]. In the next section I illustrate how I use the findings of these PPO and Coronial investigations as data.

## Materials and Methods

This article reports findings from a large research project running from 2016-2021 which sought to consider how prison oversight bodies (e.g., death investigations, (inter)national prison inspections, voluntary organizations interested in penal reform) could better prevent deaths. Initial phases of the project examined three research questions: how is prison suicide regulated?, who is regulated?, by whom is regulation undertaken? [see also (6)]. Later phases of the project (2019-2021), which produced the results reported here, more specifically examined: how do the PPO (seek to) effect change in prisons following prisoner suicides?, how could death investigations have more impact on practice and safety in prisons? [see also (9)].

As part of this project, retrospective case analysis of deaths was undertaken. This involved document analysis of >100 publicly available PPO fatal incident investigation reports^3^ about prisoner suicides. On the PPO website, the “Fatal Incident reports” page provided the functionality to filter reports by category. Two filter categories were selected: “location,” under which “prison” was selected and “cause,” under which “self-inflicted” was selected. “Gender,” “age” and “establishment” were all left on their default setting of “all.” Remaining reports were sorted in reverse chronological order, with the most recent deaths being analyzed first. The sample contained deaths from 2015 to 2017. This retrospective case analysis phase of the project did not require ethical approval because it used data that were already in the public domain and did not contain any original human subject research. All documents were thematically coded and analyzed in Microsoft Word using thematic ethnographic content analysis, which conceptualizes document analysis as fieldwork and includes reflection upon document production. Ethnographic content analysis fitted the aims of exploring how death investigations could have more impact on practice in prisons and addressed the mediated nature of the data provided in PPO and Coroner reports. Reflexive and recursive movement between theme development, coding and analysis offered a systematic approach, whilst retaining flexibility to (re)develop analytical categories (47). The author worked through the documents and developed a flexible coding frame containing seven meta-themes. These included systemic hazards, wellbeing implications, blame and problematic narratives. Remanding people with very severe mental illness to prison from police and/ or court custody was an emerging analytical sub-theme within systemic hazards.

Purposive samples of analytically informative reports under each sub-theme were then triangulated through reference to Coroner's Prevention of Future Death (PFD) reports^4^ and prison Independent Monitoring Board Reports^5^ relevant to those cases. Coroner's reports were located through an internet search for the name of the deceased (per the PPO report), accompanied by “Coroner PFD report.” Where this was unsuccessful, the “State Custody related deaths” filter would be applied and the reports searched by date. Independent Monitoring Board Reports were located by searching for the appropriate year's annual report for the prison where and when the deceased was remanded. Purposive samples under emerging sub-themes were appropriate for this exploratory analysis using relatively novel data sources, however the samples and analyses are not representative (48) of e.g., all people remanded with very severe mental illness and questionable capacity. Significantly, analysis was led by the PPO findings so the cases included here are not representative of the total number of people who died in prison having been remanded with severe mental illness. Included cases represent those for which the PPO report was available at the fixed point in time in (49) when data were collected, and for which that PPO report explicitly acknowledged severe mental illness upon remand. PPO and Coronial findings are partial and mediated, yet deserve much more scholarly analysis as they are easily accessible, public sources offering underexploited opportunities to identify learning that could reduce the harms and costs of prison suicide. Hopefully the data sources and analytical sub-theme presented in this article will act as a springboard to underpin further research and applications to policy and practice, in order to ultimately reduce “misplaced” psychiatric patients in the criminal justice system, improve prison safety and reduce psychiatric morbidity.

## Results

These four case studies: Lewis Francis, Jason Basalat, Sarah Reed and Dean Saunders all died by their own hand, having been remanded to prison despite being very mentally unwell at the time of their alleged offense and transition into prison. Jason Basalat survived in prison for <24 h, having been remanded for his own protection.

### Mr Lewis Francis (d. 2017)

Mr Lewis Francis died at HMP Exeter, England on 24^th^ April 2017 at 20 years old. He was remanded to prison having transitioned through police and court custody. His alleged crime was committed whilst acutely psychotic on 15^th^ February 2017, subsequent to which, whilst detained by Avon and Somerset Police at Bridgwater Custody Suite, he was deemed unfit to be interviewed due to his continuing psychosis (50, 51). A mental health assessment in police custody on 16^th^ February 2017 concluded that Mr Francis “lacked the mental capacity to engage with the criminal justice system” [(50): p. 6]. It is unclear how this assessor's judgment aligns with legal fitness to plead provisions or liaison and diversion processes. Coroner Rheinberg and the PPO noted that Mr Francis' illness and lack of capacity was unequivocally identified *at the first stage* of his detention transition process (50, 51). According to Coroner Rheinberg [(51): p. 1], whilst in police custody, Mr Francis' condition “mandated a transfer to a medium secure mental health hospital for an assessment and/ or treatment under section 2 and / or 3 of the Mental Health Act 1983”. Nevertheless, from Bridgwater police Custody Suite “no ready facility existed for such a transfer” [(51): p. 1]. As such, although unfit to be interviewed in police custody, and in the absence of a revised assessment that established renewed capacity, Lewis Francis was apparently fit to transition into prison and was “remanded in custody to HM Prison Exeter from where he was not transferred to a medium secure mental health hospital” [(51): p. 1].

HMP Exeter is a local prison serving the courts of the South West. On the day of Mr Francis' remand, 17^th^ February 2017, a doctor in the receiving prison recorded that he was “agitated and distressed […], displayed evidence of thought disorder and his behavior was severely disinhibited” [(50): p. 6]. This doctor recorded “serious concerns as to *whether prison was an appropriate environment for Mr Francis* […] (which) the pre-custody psychiatric report […] echoed […] and did not give a clear reason why Mr Francis was processed through the criminal justice system” [(50): p. 6, emphasis added). The prison doctor “asked for an urgent mental health assessment to be carried out and prescribed diazepam (for anxiety disorders) for 3 days” [(50): p. 6]. The doctor's requested “urgent” mental health assessment was not carried out before Mr Franci's self-inflicted death, which occurred more than 9 weeks after his transition to HMP Exeter on remand.

Mr Francis was therefore imprisoned between 17^th^ February and 24^th^ April 2017 without a criminal conviction, whilst acutely unwell and without having had a mental health assessment. In the interim, Mr Francis: was placed on resource-intensive constant watch from prison staff for at least eleven days, had 29 face to face interventions from healthcare staff and was the subject of at least seven multidisciplinary enhanced case reviews under the Assessment, Care in Custody and Teamwork system used to support prisoners at risk of suicide and self-harm [(50): p. 6-7]. On 24^th^ February 2017, Mr Francis told a prison psychiatrist that he “believed that his current experiences had alien involvement, that he was being baked alive, and that he believed he was a ghost,” and the psychiatrist recorded that Mr Francis “had suffered a psychotic episode and that his level of insight into his beliefs fluctuated greatly” [(50): p. 7]. On 27^th^ February 2017, Mr Francis told a consultant psychiatrist and learning disabilities nurse that he “felt he was burning up inside, suffered from disturbed sleep, heard voices and was distressed as he did not know how to deal with these experiences” [(50): p. 7]. In addition to Mr Franci's distress, uniformed prison staff are not trained mental health professionals and the level of staff resource required by the attempts to manage Mr Francis is likely to have negatively affected the regime for other prisoners and staff at HMP Exeter.

Coroner Rheinberg highlighted the lack of transfer mechanisms from police custody as a matter of concern extending at least across the South West [(51): p. 1-2], finding that there was “no mechanism for the ready transfer of a person in police custody within the police areas of Devon and Cornwall, Avon and Somerset, Wiltshire and Gloucestershire from police custody to a medium secure mental health facility for assessment / treatment.” The Coroner recommended that action should be taken “so as to provide for the transfer of mentally ill prisoners direct from police custody” in the South West, noting that “such an arrangement exists in the West Midlands where a Memorandum of Understanding has been developed and agreed between relevant agencies” [(51): p. 2]. Establishing robust mechanisms through which all police officers and local custody suites can transfer detainees charged with a serious crime to mental health facilities requires urgent *nationwide action* rather than local resolution. Conflicts between the definition of diversion adopted by liaison and diversion and the CPS notwithstanding, it is also imperative that police custody suites are trained and equipped to highlight that detainees are mentally ill to the CPS, given recent findings that “information from the police to the Crown Prosecution Service about an individual's mental health needs is often not clearly communicated or transferred at all, even when it is identified” [(25): p. 4, see also p. 8].

In the process of securing a mental health assessment and/ or treatment facility for a detainee who is very mentally unwell in police custody, each criminal justice transition from police, court and prison custody is a potential diversion point. Both establishing multiple safeguards and highlighting each missed opportunity after failures such as deaths is beneficial. However, it is problematic that neither the Coronial nor the PPO report into Mr Franci's death considered the transition from police to court custody as an additional potential point of diversion into mental health assessment. It is unclear from both the PPO and Coroner's reports which court remanded Mr Francis. If Mr Francis was remanded by Magistrates, which is likely, the CJJI's recommendation that “Magistrate's courts should have additional powers to bring them in line with Crown Courts, including being able to remand on bail someone for assessment without a conviction under section 35 of the MHA and to remand them for treatment under section 36 of the MHA” is relevant [(25): p. 23]. It may also be valuable for Magistrates to be made aware of the risks of remanding people with very severe mental illness to prison. If the Crown Court made the decision, more awareness of their power to remand a defendant to hospital for a medical assessment or treatment under S. 36 of the *Mental Health Act 1983* is required nationally. Additionally, there is a need to mobilize evidence such as that presented in this article in order to extend capacity for mental health assessments and treatment. Although not referenced in these four case studies, the CJJI [(25): p. 4] recently highlighted that “judges expressed frustration and concern that defendants with mental ill-health sometimes had to be remanded in prison to await an assessment or receive other support due to a lack of appropriate alternatives.”

In contrast to the Coroner's findings, the PPO's report of January 2018 stated unambiguously: “we consider that staff at Exeter could not have predicted that Mr Francis intended to take his own life on 24 April and, therefore, could not have prevented his actions” [(50): p. iii]. There is, however, rigorous evidence that individuals with psychotic disorders and delusional-like experiences are at increased risk of suicide compared to the general population (52), and there are high levels of psychiatric morbidity amongst people in prisons (53). Whilst the PPO may have had very good reasons for their judgment, their underpinning evidence base and its application to this case is unfortunately not stated. As such, it would be useful for the PPO to transparently explain the basis upon which they judge a death to (not) be predictable or preventable.

The PPO made no recommendations in Mr Francis' case, on the basis that prison staff had referred him for an “urgent” mental health assessment and that HMP Exeter had complied with local and national prison policies in this case. Although the PPO report notes that “Mr Francis' mother wanted to know what consideration, if any, was given to sectioning her son under the Mental Health Act,” the PPO neither engaged with this substantive issue nor addressed its absence in their report [(50): p. 3]. The PPO's lack of recommendations problematically reinforces the notion that remand to prison as an acceptable means of (eventually) transitioning into secure hospital, fails to challenge the lack of diversion from police or court custody and fails to highlight the delay of more than 9 weeks for Mr Francis to have an “urgent” mental health assessment as being problematic. Although the Coroner did later address some of these points, the utility of these inconsistent PPO and Coronial investigation outcomes is questionable and risks creating confusion for the services and bereaved families involved, as well as leaving risky practices and inadequate service provision unaddressed.

### Mr Jason Basalat (d. 2016)

“*Mr Basalat was arrested on 09/12/16 […] after he had grabbed the steering wheel of a coach. […] He was charged and remanded by Northamptonshire Magistrates Court. […] Bail was refused for his own protection. […] He was found hanging from a bed frame […] in his cell” [(**54**): p. 1]*.

Mr Jason Basalat died at HMP Woodhill, England on 11^th^ December 2016 at 52 years old.

After grabbing a coach steering wheel on 9^th^ December, Mr Basalat was arrested and told the police that: he had just been released from hospital, was a paranoid schizophrenic […] and believed people on the coach with guns and bombs were trying to kill him, […] he panicked and tried to stop the coach to get off [(55): no pagination]. Mr Basalat was diagnosed with schizophrenia and was “behaving in a bizarre manner” at the site of his alleged offense and in police custody, yet transitioned from police custody to court [(54): 1]. At court Mr Basalat's solicitor was informed that a mental health assessment was not possible due to it being Saturday morning (54). Mr Basalat was then remanded to HMP Woodhill “for his own protection” on 10^th^ December 2016 but survived in prison for <24 h (56).

In 2016, HMP Woodhill was both a local prison and a high security prison holding more than 800 men. Upon Mr Basalat's reception on 10^th^ December, staff “described his behavior as “bizarre” and noted that “he had defecated in the induction waiting room,” [(56): p. 5] but “did not understand that defecating on the floor was unacceptable” [(56): p. 9]. An officer described Mr Basalat as “not ‘compos-mentis,”' whilst another recorded that “Mr Basalat asked him when they would go to the pub.” [(56): p. 9]. Mr Basalat “refused to co-operate” at an initial health screen with a mental health nurse, who then “suggested that he should share a cell because of his mental health issues” [(56): p. 9]. However, Mr Basalat's cell sharing was unsuccessful and his cellmate was moved that evening after telling a member of prison staff, whilst “very distressed, scared and physically shaking,” that Mr Basalat was “crazy […] (and) had tried to light a fire” [(56): p. 9]. During Mr Basalat's short time in prison he had multiple confused exchanges with prison staff, for example, on 10^th^ December telling an Operational Support Grade (OSG) that “he could not find his phone and had lost his coat […]. During the night, Mr Basalat asked for his cell door to be left open as he needed his coat and had to go for a walk.” [(56): p. 10]. At 06.25 on 11^th^ December, “Mr Basalat was confused and told the OSG he had to leave for work at 9.00am, and needed to buy an alarm clock;” and at 7.15 am Mr Basalat “thought he was going to be kidnapped” and told an officer that he “knew they were coming for him” [(56): p. 10]. He was sadly pronounced dead at 09.35 that morning.

After the inquest in November 2017, Mr Basalat's mother Catherine Thomas said:

“Jason should never have been locked up in a prison cell […] and left unattended considering his state of mind. […] He was clearly not in a good place and was mentally very, very vulnerable. I have heard from the evidence that some things were not available because it was a weekend. […] If Jason had been treated properly and by the correct professionals, he would without a doubt still be here. This is beyond distressing. […] I pray to God that this never happens again to any family, ever. Lessons need to be learnt and sadly should have been previously.” [(55): no paginatio].

As argued above, in the process of securing an appropriate assessment and treatment in a (secure) mental health facility, each transition between police, court and prison custody is a potential failure point. Both establishing safeguards at multiple points in this process and then highlighting them after failure is beneficial. In Mr Basalat's case, it is problematic that neither the Coroner nor the PPO report questioned his transition from police to court custody. As such, not only are robust mechanisms required nationally to facilitate transfer of ill detainees from police custody to mental health facilities, but greater awareness of this point of failure is potentially required amongst police, criminal courts, coroners and the PPO. In this case, Coroner Osborne overlooked the transfer to court but highlighted that, “at court, consideration should have been given as to the most appropriate place for the deceased to be held or to receive a mental health assessment” [(54): p. 2]. However, this is a vague provision which misunderstands that Magistrates courts do not currently have the power to remand someone “for treatment under section 36 of the MHA” [(25): p. 23]. Practitioner training and increased mental health assessment and bed capacity are also likely required. Mr Basalat's case is particularly sad because Northamptonshire Magistrates Court refused his bail “for his own protection” [(54): p. 1], facilitating his remand to a prison environment that “is not a safe place to wait for a hospital bed” [(26): p. 6; see also p. 20] and that records high levels of psychiatric morbidity (53).

The PPO report of December 2017 focussed on information recording and sharing in their recommendations [(56): p. 5]. The PPO appear to tacitly focus their investigation on practices within prison, but it would be useful to transparently state this and consider that failing to highlight the lack of diversion from police or court custody serves to perpetuate the remand of people to prison rather than hospital, and also risks (mis)assigning blame for deaths to prison staff who are not trained to manage people with serious mental illnesses. Moreover, the PPO's report stated unambiguously that, although Mr Basalat was remanded for his own protection with a known psychiatric diagnosis of schizophrenia whilst apparently having a relapse and active delusional-like experiences (52, 53), “there was no evidence that he was at imminent risk of suicide or self-harm” [(56): p. iii]. Nevertheless, in the case of *Keenan v. the United Kingdom (2001)* (application 27229/ 95), the European Court of Human Rights dismissed a claim under Article 2 (right to life) of the European Convention on Human Rights/ *Human Rights Act 1998* in the UK because Keenan's record contained no formal diagnosis of schizophrenia, a condition in which the risk of suicide was well known and high, hence the authorities could not have known that he was an immediate suicide risk. Again, it would be very useful for the PPO to state the basis for their conclusion, particularly with reference to the *Keenan* judgment, and to consider the utility of their tacitly prison-focussed investigation producing inconsistent outcomes against the inquest.

### Ms Sarah Reed (d. 2016)

“*This is a particularly troubling case of a seriously unwell woman being held in a prison setting which, despite commendable efforts by some staff, proved incapable of keeping her safe.” [(**57**): p. iii]*.

Ms Sarah Reed died at HMP Holloway, England on 11th January 2016 at 32 years old. She “suffered from serious mental health problems” [(57): p. iii] and was remanded to prison from the Inner London Crown Court on 14^th^ October 2015 “solely for the purpose of obtaining […] reports on her fitness to plead and stand trial” for an alleged offense [(58): p. 2]. Whilst on bail for the alleged offense, she had not attended two psychiatrist appointments arranged to facilitate assessment of her fitness to plead and was remanded to HMP Holloway so the Court could obtain these reports [(58): p. 2]. The Crown Court judge stated “I can't see any way these reports will be prepared whilst the defendant remains on bail” [(58): 2]. By the time that Ms Reed died, 3 months after entering prison, only one report had been obtained and “a second report was due on 15 January 2016. No date had been fixed by the Crown Court for a hearing to determine […] her fitness to plead” [(58): 2]. From 5^th^ January 2016 in prison, Ms Reed's “mental health deteriorated further and her behavior became erratic and unpredictable. She spent long periods shouting, chanting and making noises in her cell” [(57): p. 1].

At inquest in July 2017, Coroner Thornton highlighted that: “had the Court obtained the psychiatric reports on fitness to plead earlier, the Court may well have imposed a hospital order (with or without a restriction order) under section 5(2)(a) of the Criminal Procedure (Insanity) Act 1964. The two necessary requirements would have been easily satisfied: the reports were to find her unfit to plead and she had admitted the act charged” [(58): p. 3]. Coroner Thornton noted that it was unclear who took responsibility for obtaining the psychiatric reports between the Court and the prison, highlighting the need to “consider whether the procedures for obtaining and providing psychiatric reports on the issue of fitness to plead […] are sufficiently timely, sufficiently robust and sufficiently well-managed” [(58): p. 4]. However, this provision is vague and the Coroner was apparently still endorsing the risky use of imprisonment for people with severe mental illness for the purpose of obtaining psychiatric reports, albeit in a more “timely” manner. The Coroner then sent his report to “MoJ; HMCTS; HMPPS; CNWL^6^” [(58): p. 4], with the inclusion of multiple organizations meaning that there was no clear ownership of the action. Although the coroner did specify that “HMCTS may wish to consider whether the courts have sufficient control over the process […] so as produce reports sufficiently promptly” [(58): p. 4], the roles of the other three actors remain unclear in the action. In contrast to the cases of Mr Francis and Mr Basalat, Ms Reed's case indicates a need for Crown Court judges (rather than Magistrates) to be made aware of the risks of remanding people with severe mental illness to prison and for alternative hospital transfer mechanisms and beds to be available. It is striking that the Coroner did not explicitly consider whether the Crown Court could have ordered the transfer of Ms Reed to a mental health facility rather than prison (e.g., under section 36 of the Mental Health Act 1983) such that reports on her fitness to plead could have been obtained, again indicating a potential need for awareness of this power and/ or increased assessment and bed capacity.

The PPO report of January 2017 stated “it is essential that lessons are learned from Ms Reed's tragic case” (PPO, 2017: p. iii). The PPO or Coroner were clear that Ms Reed's death was deeply problematic, but unfortunately neither engaged with the substantive issue: that a very ill woman was remanded to prison in the first place. Neither the PPO nor Coroner explicitly considered whether HMP Holloway could have been reasonably expected to obtain psychiatric reports on Ms Reed's fitness to plead and keep her safe. It is perhaps also significant that the closure of HMP Holloway was suddenly announced on 25^th^ November 2015^7^, during Ms Reed's remand there, although neither the PPO nor Coroner engaged with the implications of this announcement for staff practice and service delivery.

Finally, the PPO report noted that “Ms Reed's family had a number of questions,” which included “was it appropriate for Ms Reed to be in prison or should she have been moved to a secure hospital?” [(57): p. 4]. The PPO report did not, however, engage with this question, highlight its relevance in the case nor explain whose remit it was to consider whether it was appropriate for Ms Reed to be remanded to prison for the purposes of obtaining reports on her fitness to plead and then to be kept in prison when her condition began to deteriorate.

### Mr Dean Saunders (d. 2016)

“*Mr Saunders was acutely mentally ill and all those involved in his care agreed that prison was not an appropriate place for him” [(**59**): iii]*.

Mr Dean Saunders died at HMP Chelmsford, England on 4th January 2016 at 25 years old. He was remanded to prison on 18^th^ December 2015 after being “identified as seriously mentally ill and need(ing) hospital treatment” in police custody and believing “his family were involved in a conspiracy against him” [(59): p. 6]. In police custody on 16^th^ December 2015, the on-call psychiatrist recommended that Mr Saunders be assessed for admission to a forensic mental health ward. However, “Rochford Hospital could not offer a bed for Mr Saunders,” who then transitioned from police custody to Basildon Magistrates' court on 18^th^ December and was remanded to HMP Chelmsford, a local prison that takes prisoners directly from the courts [(59): p. 10]. This indicates that mental health bed numbers are insufficient, and highlights the need to challenge the practice of using remand to prison as a substitution for beds in mental health facilities.

In February 2017, the Coroner issued a Prevention of Future Deaths report which explicitly raised, as a matter of concern, that South Essex Partnership Trust (healthcare, SEPT) and NHS England consider “whether the transfer of individuals such as Dean to prison is indeed ‘best practice,' taking into account the consequent delay in transfer and the suitability of the prison environment for mentally disordered individuals” [(60): p. 4]. The Coroner also directed SEPT to establish a mechanism to transfer “mentally disordered people from police custody” to hospital, rectifying the “admitted lacuna in the SEPT admissions protocol (which) […] does not allow for the transfer of *any* individual from police custody” [(60): p. 4]. As such, the Coroner had made a local and national healthcare recommendation to (re)consider the imprisonment of mentally unwell people and a local recommendation to establish a police custody–hospital transfer mechanism. Yet, as highlighted by the later case of Mr Lewis Francis in the South West region, such transfer mechanisms are *required nationally*.

On 20^th^ December, Mr Saunders was at HMP Chelmsford and under constant watch by Officers. The evening officer noted that Mr Saunders was:

“agitated and tearful and kept repeating ‘this is a game'. […] He thought he was under surveillance, that one of the nurses had a microphone in her hair, and that another member of staff had a camera in her glasses.” [(59): p. 13].

Despite acknowledging the unsuitable nature of imprisonment for Mr Saunders, none of the PPO's eight recommendations [(59): p. 1] engaged with his remand. The most relevant recommendations directed the Head of Healthcare at HMP Chelmsford to “ensure that there is an established process, in line with national guidance, which healthcare staff understand and follow, to transfer prisoners to hospital under the Mental Health Act, within 14 days where possible” [(59): p. 6]. However, the case of Mr Basalat, who survived in prison for <24 h, reminds us that remanding people with very severe mental illness transitioning into prison in the first place is a risky practice, and that imprisonment is not a safe or *acceptable pathway into (secure) healthcare*. The PPO also directed the NHS England East of England Area team to “ensure that psychiatric services commissioned for prisoners at HMP Chelmsford are sufficient to meet their needs and reflect community provision” [(59): p. 16]. As such, the PPO again tacitly accepted the imprisonment of vulnerable people with severe mental illness, deflecting attention from essential questioning of the imprisonment of people with severe mental illness and focusing instead on constrained debates around transfer processes and times, and the commissioning of psychiatric services in prison. Moreover, the PPO were critical of prison staff suicide and self harm management [(59): p. 6], essentially deflecting attention for Mr Saunders' inappropriate imprisonment onto prison staff. In turn, the PPO overlooked that these staff were managing a very unwell man for whom prison was unsuitable, and were working in an enduringly chaotic, low staffed prison, managing problems which were entirely “beyond the(ir) control,” including “dilapidated” Victorian buildings, “Government budgetary cuts […] reflect(ing) a much lower number of officers” and the “apparent lack of secure mental health accommodation available outside of Prison” [(61): p. 5].

## Discussion

I have argued that prison is too frequently (tacitly) accepted or utilized as a “place of safety” or pathway into (secure) healthcare for people with very severe mental illness. I have highlighted the particular risks of remand through four case studies of people with very severe mental illness at the time of their alleged offense and remand to prison who went on to take their own lives in prison. It is crucial that scholarship, health and justice practice and death investigations challenge the transitions of people with very severe mental illness through police and court custody into prison *at all*, given that “the prison is not a safe place to wait for a hospital bed” [(26): p. 5-6]. Crises such as prison suicide involve complexity, but those individuals and organizations directly involved in the crisis and investigating it afterwards (which includes scholars) can affect how the crisis unfolds, by their actions or omissions (62). As such, groups including PPO investigators, Coroners and scholars must think productively “about crises in ways that highlight their own actions and decisions as determinants of the conditions they want to prevent” [(62): p. 316]. Prison is not, and cannot be a substitute for or safe pathway into secure healthcare. Questioning the appropriateness and highlighting the implications of people with severe mental illness at the time of their offense and remand entering prison *in the first place* serves to problematise recent scholarship on prison mental health, including e.g., spaces of potentiality for prison mental health care (63); the potential of trauma-informed practice in prisons (64); transfer times from prison to high and medium security psychiatric care (65); and the “success” of liaison and diversion services (30, 31). Although prisoners may develop (more severe) mental illness whilst in prison, and should of course be provided with appropriate care and promptly transferred to hospital where required, this scenario should not be equated with people with very severe mental illness at the time of their alleged offense and remand entering prison in the first place.

A significant limitation of this study is that analysis was led by the PPO findings. As such, the cases four cases discussed here are not representative of the total number of people who died in prison having been remanded with severe mental illness between January 2016 and April 2017. Included cases represent those for which the PPO report was available at the fixed point in time in 2019 when data were collected, and for which that PPO report explicitly acknowledged severe mental illness upon arrest and remand to prison. It would be valuable to consider a larger sample of cases over a longer period of time, along with further data sources that could develop the arguments presented here. Further data sources could include interviews with health professionals in police custody suites and prisons about the frequency of people with severe mental illness at the time of their alleged offense being remanded to prison; interviews with Magistrates and Crown Court judges about the barriers to using their powers to remand to hospitals; Multidisciplinary Team Reviews of the points of failure in these four cases and further identified similar cases; and brainstorming of template recommendations to guide PPO investigators and Coroners and increase the efficacy of their reporting.

My findings have multiple implications. Fundamentally, challenging the apparent cross-institutional (acceptance of the) use of prison as a “place of safety” and/ or acceptable pathway into secure hospital is important. Seriously mentally unwell people being remanded to prison is a particularly risky practice requiring urgent multiagency action, as summarized below. My principal finding is that police custody suites nationally require robust mechanisms through which very mentally ill people charged with a serious crime can be transferred to hospital for treatment and or assessment under Section 2 and / or 3 of the *Mental Health Act 1983*, as opposed to the use of arrest and criminal justice pathways. Recent assessment indicates that police officers recognized “that more serious investigations could progress in parallel with mental health treatment or could be put on hold pending such treatment” [(25): p. 58]. There is, however, a longstanding lack of availability of mental health facilities to support assessments and inpatient treatment. As such, it would be valuable for scholars and death investigators to amplify the CJJI recommendation that “the Department of Health and Social Care, NHS England and Improvement and Welsh Government should: ensure an adequate supply of medium and high secure beds” [(25): p. 12]. This would apparently be beneficial both ethically and practically (given the harms and costs of prison suicide and the potential for “clustering”).

Next, in the process of securing a mental health assessment and/ or treatment facility for a detainee who is very mentally unwell in police custody, each criminal justice transition from police, court and prison custody is a potential diversion point. Both establishing multiple safeguards and highlighting each missed opportunity after failures such as deaths would be beneficial: detainees with severe mental illness could be transferred to hospital from *both* police and court custody. Again, I amplify the CJJI's recommendation that “Magistrate's courts should have additional powers to bring them in line with Crown Courts, including being able to remand on bail someone for assessment without a conviction under section 35 of the MHA and to remand them for treatment under section 36 of the MHA” [(25): p. 23]. I have argued that death investigators, Magistrates and potentially Crown Court judges nationally require more awareness of powers and omissions under the *Mental Health Act* 1983 enabling courts to transfer detainees charged with a serious crime to mental health facilities for medical reports, along with robust mechanisms to facilitate this in practice. Relatedly, education to create awareness of the risks of remanding people with very severe mental illness to prison is required, along with awareness of the functions that can reasonably expected from prisons (which are not facilities for mental health assessment), potentially for Magistrates, Crown Court judges, PPO investigators, Coroners and staff in police custody suites.

More specifically, the PPO should transparently explain the basis upon which they judge a death to (not) be predictable or preventable. The PPO should also clarify and make explicit that their remit involves examining compliance with local and national prison policies whilst the deceased was in prison, and do this throughout their Terms of Reference, investigation reports and communications with bereaved families. It would be beneficial for the PPO to give clear examples about the limits of their remit to all stakeholders from the earliest opportunity, to repeat these consistently throughout public and private communications and to explicitly include them in death reports. Within this point, the PPO could also usefully consider whether their current remit is defensible as their practice of examining only prison compliance with local and national prison policies does not easily align with their stated position as “wholly independent” nor terms of reference: “to execute fair and impartial investigations […] without fear” [(46): p. 1] and “to investigate the circumstances of the deaths of prisoners (and) to examine whether any change in operational methods, policy, practice or management arrangements would help prevent a recurrence” [(46): p. 8]. From reading these provisions, it would be reasonable to assume that being imprisoned when acutely mentally ill is central to the circumstances of a death, and families have repeatedly questioned the practice of remand without public answer from the PPO.

Finally, I have highlighted disparities between the PPO and Coroner's findings in the same cases. This indicates that the PPO should *reconsider the (lack of) relationship between their findings and recommendations and those made by Coroners* and other prison and detention oversight bodies (such as the national Inspectorate and local Independent Monitoring Boards). PPO draft reports are shared with stakeholders far more rapidly than Coroner's inquest findings are produced (44). Very few stakeholders are likely to read and analyse full PPO reports beyond their “headline” findings and recommendations. Significant inconsistencies between PPO and Coronial findings can cause confusion for the services and bereaved families involved, and perhaps indicate that the PPO have not departed sufficiently from the practices of the Prison Service's own investigators who undertook this function before 2004 (6, 9). The disparity between PPO and Coronial findings also counters the apparent “problem of implementation” in prison oversight, where the apparent limitation is simply that “recommendations are not implemented” and instead highlights the need to *question what overseers recommend, based on which evidence*. Stronger relationships between various overseer's findings could amplify calls for the systemic implementation of core recommendations (13), and challenge problematic practices involving multiple health and criminal justice agencies, such as the remanding of people with very severe mental illness into prisons.

My focus intersects with further systemic issues which are beyond the scope of this article but also deserve further multidisciplinary analysis. These include (lack of) mental health services in prison; prisoners who become (more) mentally unwell whilst in prison; transfer and remittal between prison and hospital; the prevalence of, e.g., (undiagnosed) learning disabilities, autism, attention deficit hyperactivity disorder, Fetal Alcohol Spectrum Disorder and acquired brain injuries amongst prisoners; and the harms of mental health detention. Future work could valuably consider the extent to which my arguments about the limits of criminal responsibility and exclusions on whom can be imprisoned as applied in practice relate to prisoners with e.g., traumatic brain injuries and addiction.

## Data Availability Statement

The original contributions presented in the study are included in the article. The data that support the findings of this study were derived from the following resources available in the public domain are: https://www.ppo.gov.uk/document/fii-report/; https://www.judiciary.uk/subject/state-custody-related-deaths/; https://www.imb.org.uk/. Further inquiries can be directed to the corresponding author.

## Ethics Statement

Secondary data available in the public domain were exclusively used. Written informed consent was not obtained from representatives of the deceased for the publication of identifiable data included in this article.

## Author Contributions

PT completed all the data collection, analysis, and writing.

## Funding

This work was supported by UK Research and Innovation [Grant Number: MR/T019085/1].

## Conflict of Interest

The author declares that the research was conducted in the absence of any commercial or financial relationships that could be construed as a potential conflict of interest.

## Publisher's Note

All claims expressed in this article are solely those of the authors and do not necessarily represent those of their affiliated organizations, or those of the publisher, the editors and the reviewers. Any product that may be evaluated in this article, or claim that may be made by its manufacturer, is not guaranteed or endorsed by the publisher.

## Footnotes

^1^Outside Scandinavia, evidence of intention is crucial to suicide classificiations. For cases where capacity to form intent is questionable, ‘self-inflicted death' is arguably more appropriate, to encompass intentional and accidental deaths. However, intention in completed self-inflicted deaths is often unclear, and confused and mixed intentions can be seen in (attempted) self-inflicted deaths and self-harm incidents (1).

^2^ https://www.gov.uk/government/statistics/safety-in-custody-quarterly-update-to-june-2021

^3^ https://www.ppo.gov.uk/document/fii-report/

^4^ https://www.judiciary.uk/subject/state-custody-related-deaths/

^5^ https://www.imb.org.uk/

^6^The Ministry of Justice; HM Courts and Tribunal Service; HM Prison and Probation Service; Central and North West London NHS Trust.

^7^https://www.gov.uk/government/speeches/prisons-announcement (accessed January 25, 2022).
